# Postpartum Hemorrhage and Tranexamic Acid: A Literature Review

**DOI:** 10.7759/cureus.38736

**Published:** 2023-05-08

**Authors:** Satvika Anaposala, Pavan Kumar Reddy Kalluru, Ernesto Calderon Martinez, Sushmitha Bhavanthi, Chethan Raj Gundoji

**Affiliations:** 1 Obstetrics and Gynecology, Tata Motors Hospital, Jamshedpur, IND; 2 Obstetrics and Gynaecology, Sri Venkateswara Medical College, Tirupati, IND; 3 Biomedical Informatics, Universidad Nacional Autónoma de México, Ciudad de Mexico, MEX; 4 Gynecology, Government Medical College, Nizamabad, Nizamabad, IND; 5 Internal Medicine, Government Medical College, Nizamabad, Nizamabad, IND

**Keywords:** postpartum hemorhage, tranexamic acid, uterine atony, cesarean section (cs), postpartum bleeding

## Abstract

This review aims to explore the postpartum hemorrhage (PPH) burden and the efficacy of prophylactic tranexamic acid (TXA) in PPH and recent indications of TXA. A comprehensive review of the literature was conducted using a combination of Medical Subject Headings keywords including “Postpartum haemorrhage,” “Tranexamic acid,” and “Cesarean section.” PPH has been explored for epidemiology, risk factors, and pathophysiology in the first part of the article. Recent indications of TXA, obstetric indications, and the role of TXA as prophylaxis for PPH are discussed in the second part of this article. TXA has many indications apart from obstetric indications and shows a significant effect in controlling bleeding. Furthermore, TXA is more efficient in preventing PPH if administered during the final stage of labor and is a valuable option for managing obstetric bleeding.

## Introduction and background

Postpartum hemorrhage (PPH) is traditionally described as blood loss of more than 500 mL with a vaginal birth or more than 1,000 mL with cesarean delivery. Worldwide, more than 500,000 women pass away every year from pregnancy and delivery-related causes. A significant portion of mortality and morbidity, including severe anemia, the need for blood transfusions, hospital stays, and infections, are caused by PPH. Only a small portion of all maternal deaths are caused by PPH in high-risk individuals. The majority of problems are unpredictable and occur in people with no risk factors. In a previous study, 9.2% of 1,620 women in rural India reported having PPH. In most cases, no difference was noted between maternal or sociodemographic characteristics between women with PPH and those without [1]. Although cesarean section (C-section) deliveries had a lower incidence of early PPH (occurring within 24 hours of delivery) than vaginal deliveries, the former is a major surgery and results in more blood loss [1]. Therefore, it is crucial to effectively and efficiently limit blood loss, which explains the need for this research. Pregnant women aged between 15 and 49 years are considered to be a vulnerable group for anemia [2]. Anemia prevalence has barely changed in the 10 years among women, with the same trend seen in men. C-section rates have increased in different areas of the world, increasing mortality in high-risk anemic groups [3]. It is crucial to minimize bleeding in lower-segment cesarean sections (LSCSs) for decreasing maternal mortality due to bleeding [4].

Tranexamic acid (TXA) is an antifibrinolytic with a good safety profile in LSCS by maintaining satisfactory postoperative hemoglobin as well as hematocrit values. The WOMAN trial proposed the use of intravenous TXA early in addition to standard care for women as the antifibrinolytic effect of TXA in the third stage of labor makes this drug a safe and effective option. An activated fibrinolytic system may last up to 10 hours postpartum, which causes more bleeding, and TXA can be a valuable option in this condition [5-7].

Pharmacological management is essential in treating PPH, along with surgical and radiological interventions. Uterine atony is a major cause of PPH, and oxytocin is recommended as the first-line treatment. Other options, such as intravenous ergometrine, intramuscular carboprost, and misoprostol, are also available. TXA is a pro-hemostatic drug that offers a complementary biochemical hemostatic effect along with oxytocin. Previous research has shown that TXA is productive in lowering blood transfusion requirements in anemic women scheduled for a C-section. The use of TXA is empirically thought to be more beneficial than blood transfusion [7,8]. This study aims to investigate whether preoperative administration of TXA can help decrease blood loss in PPH.

## Review

Postpartum hemorrhage

Obstacle

The definition of PPH is not precise and requires some insight. A loss of greater than 500 mL of blood after vaginal delivery or greater than 1,000 mL after a C-section is defined as PPH. If the loss occurs within a day, it is termed early or primary PPH, whereas late or secondary PPH refers to blood loss that occurs after one day [9]. This article primarily studied early PPH. Blood loss estimates at birth are subjective and imprecise. Moreover, the diagnosis of PPH is retrospective, and aspects related to defining PPH are helpful for science and research but not in the real setting.

Epidemiology

PPH is the most common cause of maternal mortality [10]. PPH and associated complications are dangerous for all women who carry a pregnancy over the 20-week mark. Even though the mortality rates have decreased significantly in the industrialized world, PPH continues to be the prominent cause of maternal death in other parts of the world [11]. Between 1990 (8.0 deaths per 100,000 live births) and 2019 (20.1 deaths per 100,000 live births), maternal mortality rates in the United States increased three times [11]. Studies show that PPH continues to be the major reason for maternal morbidity and mortality [12]. In 2009, PPH contributed to slightly over 10% of all maternal deaths (about 1.7 deaths per 100,000 live births) [13]. Various sources, including numerous major, randomized studies conducted in industrialized nations showed that the prevalence rate of PPH is lower when active management is utilized compared to when expectant management is used [14]. Because of the dearth of treatment options for active management in the third stage, the rates indicated above for expectant management are more likely to reflect the rising prevalence of PPH in developing countries. Several other factors are thought to play a role in the poorer outcomes of PPH in impoverished nations [15]. The first is a scarcity of experienced caregivers who can effectively manage PPH. Furthermore, the medications utilized for PPH prophylaxis in active third-stage management are also the principal agents in PPH treatment. Limited availability of these medications along with the short supply of blood transfusion services, anesthesia services, and operating capabilities play a role in poorer outcomes.

Etiology

Although PPH can be caused by various factors, the most prevalent is uterine atony, which occurs when the uterus fails to contract and retract after the baby is delivered. The biggest risk factor is PPH from a prior pregnancy, and it should be investigated thoroughly to establish the etiology and intensity. Birthweight, chorioamnionitis, lacerations, instrumentation, labor induction and augmentation, hypertensive disorders, and magnesium sulfate use have all been linked to an elevated risk of PPH [16]. Sheiner et al. reported significant risk variables using multivariable analysis in a large population-based investigation [17].

Pathophysiology

During pregnancy, the volume of maternal blood increases by around half (from 4 L to 6 L). The plasma volume increases by 50% which is more than the red blood cell (RBC) volume which increases by 18% resulting in hemodilution in pregnancy. These changes provide a reserve for blood loss after delivery and meet the perfusion requirements of the low-resistance uteroplacental unit [18]. Myometrial fibers tighten and narrow the blood arteries as the placenta separates from the uterine wall during childbirth and decrease blood loss from this low-resistance vasculature termed living ligatures or physiologic sutures [19].

The most common cause of PPH is uterine atony which usually begins within four hours of the baby’s birth. Because of the increased blood flow to these tissues, injury to the genital system during delivery causes substantially greater bleeding than in the non-pregnant condition resulting in PPH. The trauma associated with the delivery of the infant, whether spontaneous or aided vaginal delivery or cesarean delivery, can be considerable, resulting in significant soft tissue disruption and blood vessel tearing [16].

Prevention

The severity and incidence of PPH can be lowered by active intervention in the final stage of labor. Because individual risk factors do not accurately predict PPH, all pregnant women should be targeted for prevention. Active management refers to a mix of techniques, uterotonic administration (ideally oxytocin) as soon as possible after delivering the baby, early clamping of the cord and cutting, controlled traction, and applying uterine massage [20]. It is generally believed that active management with uterotonic agents such as oxytocin increases retained placenta, thereby increasing bleeding chances. However, studies have shown contradicting results and have reported evidence of the benefit of early oxytocin treatment [21].

Presentation

PPH can manifest in slow and less noticeable bleeding or more spectacular forms, leading to catastrophic loss and shock. Therefore, close observation of maternal vital signs, blood loss, and uterine tone should be part of standard postpartum care nursing procedures. Heavy vaginal bleeding is the most prevalent symptom of PPH that can quickly progress to hypovolemic shock. Blood loss can be hidden in the uterus or elsewhere, and caretakers often underestimate apparent blood loss by up to 50%. PPH can occur due to retained tissue or trauma, and uterine atony can also cause blood to collect in the uterus even after the placenta has been delivered. Prompt recognition and diagnosis of PPH are critical to managing it effectively before severe hypovolemia develops. Delay in commencing adequate care is a crucial factor in negative outcomes associated with serious bleeding and so immediate intervention must be done swiftly. Most women who give birth are healthy and can easily adjust to blood loss, which means hypovolemia symptoms may not appear immediately. Hematomas in the cavity of the abdomen, the retroperitoneum, and the lower vaginal tract can also hide traumatic bleeding. It is essential to recognize and diagnose PPH as early as possible to prevent severe hypovolemia and negative outcomes [22-24].

Contraindications

To our knowledge, there are no definite contraindications to the procedures used to treat PPH according to the literature, with the exception of situations such as non-consent, a lack of surgical expertise, or an allergy to particular drugs which are non-specific and generally noted.

Tranexamic acid

Pharmacology

**Figure 1:**
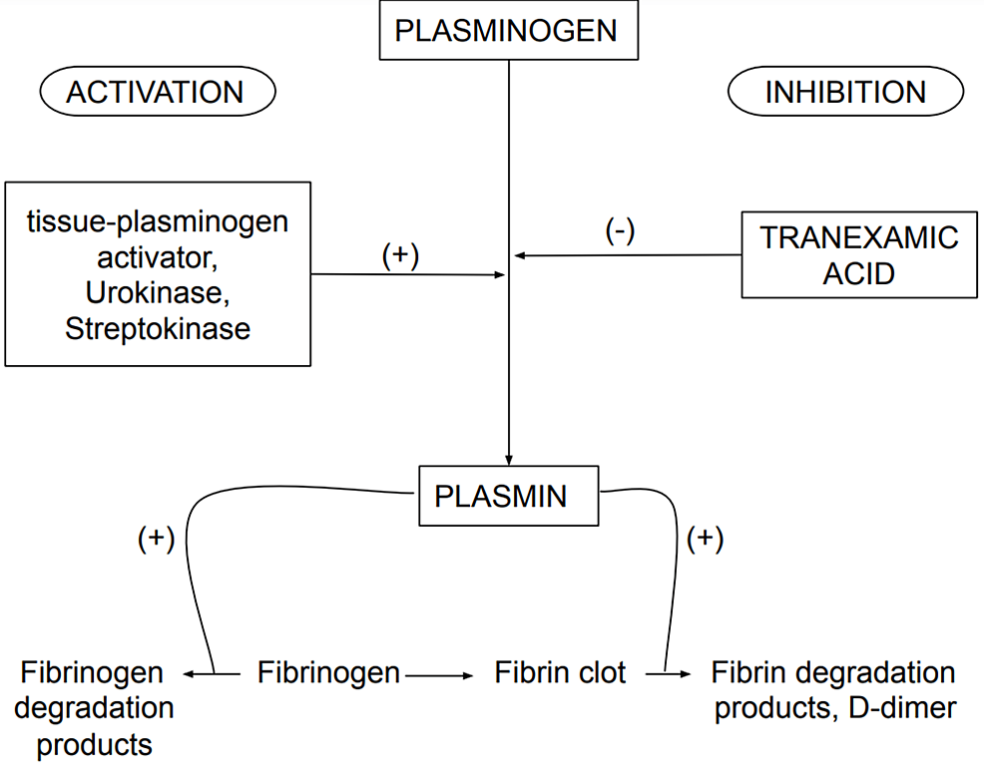
Pharmacological action of tranexamic acid.

TXA, a synthetic lysine derivative, inhibits the interaction of plasminogen with produced plasmin and fibrin and has antifibrinolytic actions by blocking lysine binding sites on plasminogen. This results in the stabilization of preformed fibrin meshwork fabricated by secondary hemostasis [25]. Figure 1 demonstrates the pharmacological action of TXA.

The bioavailability of TXA was significantly higher after intravenous and intramuscular administration compared to oral administration. Specifically, the bioavailability after intravenous administration was 100%, after intramuscular administration was 76.7%, and after oral administration was only 36.4%. The time to peak concentration was also significantly shorter for intravenous administration compared to intramuscular and oral administration. The results suggest that the intravenous and intramuscular routes of administration may be more effective for achieving therapeutic levels of TXA in the body [26].

Indications

TXA was approved by the Food and Drug Administration (FDA) for the treatment of excessive menstrual bleeding and short-term prophylaxis in hemophilia. Research has shown that TXA can also be beneficial for tooth extractions in hemophilia patients when used in conjunction with clotting factor replacements [27]. Additionally, TXA has been found to be beneficial in menorrhagia and enhance the comfort of life in patients. FDA has approved a dose of 1,300 mg orally, thrice a day, up to five days during menstruation for the treatment of excessive menstrual bleeding [28]. Off-label recent TXA usage in additional contexts is described in Table 1.

**Table 1:** Off-label usage of tranexamic acid. TXA: tranexamic acid

Authors	Type of study	Indication	Route of administration	Comments
Yang et al. [29]	Randomized controlled trial	Total knee arthroplasty	Intra-articular	Intra-articular TXA significantly improved pain and showed a significantly lower reduction of hemoglobin compared to controls
Choi et al. [30]	Retrospective analyzation	Multilevel (five levels) posterior spinal instrumented segmental fusion	Intravenous	Study data showed that the TXA considerably improved blood loss and required fewer blood transfusions
Myles et al. [31]	Randomized controlled trial	Undergoing coronary artery surgery	Intravenous	Findings showed that TXA was not related to an increased mortality risk or thrombotic complications within 30 days of surgery, but rather with a decreased bleeding risk than the placebo
Tavakoli et al. [32]	Randomized controlled trial	Patients with proven acute gastrointestinal bleeding	Three groups, namely, intravenous, topical + systemic, normal saline (placebo)	The findings demonstrated that TXA’s antifibrinolytic capabilities could help transition an urgent endoscopy into an elective operation, improving outcomes for medical professionals as well as patients
Zahed et al. [33]	Randomized controlled trial	Treatment of epistaxis by nasal packing (anterior packing)	Topical	TXA lowered bleeding and rebleeding episodes and improved emergency department times and patient satisfaction
CRASH-3 trial collaborators [34]	Randomized controlled trial	Adult patients with traumatic brain injury presented within 180 minutes of injury	Intravenous	Patients with mild-to-moderate brain injuries responded better to early treatment than to later therapy; however, patients with severe head injuries did not seem to be affected by treatment delay
Post et al. [35]	Randomized controlled trial	Adults with CT-proven spontaneous subarachnoid hemorrhage	Intravenous	Rebleeding was observed in 10% of the TXA group after randomization and before aneurysm therapy compared to 14% of patients in the control group. However, very early, short TXA treatment did not enhance clinical outcomes at six months
Kaur et al. [36]	Randomized controlled trial	Micro-needling in melasma	Topical	In the treatment of melasma, TXA appears to have assurance, and topical application in conjunction with micro-needling appears to be effective
Ockerman et al. [37]	Randomized controlled trial	Dental extraction in individuals who are on non-vitamin K oral anticoagulants	Oral	TXA minimized delayed bleeding and postoperative oral bleeding but not early bleeding in individuals
Moharamzadeh et al. [38]	Randomized controlled trial	Painless lower urinary tract bleeding	Topical	The findings of this investigation demonstrated that, while TXA had no appreciable impact on the decline in serum hemoglobin levels, it might greatly lower the volume of serum needed for bladder irrigation to clear urine
Batagello et al. [39]	Randomized controlled trial	Patients undergoing percutaneous nephrolithotomy for complex kidney stones	Intravenous	For those with complicated kidney stones undergoing the procedure, using TXA is safe and significantly lowers the requirement for blood transfusions
Hoyos et al. [40]	Randomized controlled trial	Scheduled liposculpture	Three groups, namely, intravenous, subcutaneous, and normal saline (placebo)	On both the first and fifth postoperative days, the investigators discovered that the intravenous group had a better hemoglobin level compared to the subcutaneous and placebo groups. Between the subcutaneous intervention and placebo groups, there were no statistically significant variations in hemoglobin values

Postpartum Hemorrhage Indication

As one of the prime causes of maternal deaths, PPH is an emergency condition to address. About 6% of pregnancies are complicated by PPH. TXA has been used as an additional treatment for PPH because of the related morbidity and mortality. Over 20,000 PPH patients were arbitrarily grouped to receive TXA or a placebo in the WOMAN trial. While there was no discernible change in the rates of thrombosis, the death rate from bleeding was much lower in the TXA group (1.5% vs. 1.9%, p = 0.045). The effectiveness of TXA in preventing PPH is being evaluated; however, the data are not as strong. TXA as a preventative measure during C-section is discussed in the following studies. Randomly, over 4,000 women were grouped to receive TXA or a placebo in the TRAAP trial to prevent PPH; however, there was no discernible difference in bleeding rates [41-43].

A meta-analysis including 18 randomized controlled trials (RCTs) with 1,764 women on intravenous TXA for bleeding control following C-sections and 1,793 controls on placebo was performed by Franchini et al. [44]. TXA use decreased the risk of PPH >400 mL (relative risk (RR) = 0.40, 95% confidence interval (CI) = 0.24-0.65; five trials with a total of 786 participants), severe PPH >1,000 mL (RR = 0.32, 95% CI = 0.12-0.84; five trials with a total of 1,850 participants), and the requirement for RBC transfusion (RR = 0.30, 95% CI = 0.18-0.49; 10 trials with a total of 1,873 participants).

In total, 36 RCTs involving 10,659 women undergoing C-sections were studied in a meta-analysis by Bellos et al. [45] in which TXA significantly decreased total blood loss (mean difference (MD) = 189.44 mL, 95% CI = 218.63-160.25), decreased hemoglobin drop (MD = 8.22%, 95% CI = 5.54-10.90), and decreased transfusion requirement (odds ratio (OR) = 0.41, 95% CI = 0.26-0.65).

A randomized double-blinded study on the effectiveness of preoperative TXA by Oseni et al. [46] among 244 women who were to have emergency C-sections showed an average blood loss of 414.0 mL in the selected group and 773.8 mL in the controls (t = - 16.18, p ≤ 0.01). Average postoperative hemoglobin and packed cell volume was 10.1 g/dL and 31.5% in the study group and 9.5 g/dL and 29.9% in the controls (t = 4.99, p ≤ 0.01 and t = 4.70, p ≤ 0.01), respectively.

Naeiji et al. [47] performed a placebo-controlled RCT with the aim to study the advantages of prophylactic TXA administration in elective C-sections. The study showed that TXA lowered the average blood loss by 25.3%. The rate of bleeding in excess of 1000 mL and 500 mL, as well as the requirement for blood transfusion, were considerably lower in TXA patients. Six hours following a C-section, the mean hemoglobin in the placebo group was considerably lesser than in the TXA set of 11.77 ± 0.50 compared to 11.31 ± 0.56 in the placebo. Intravenous TXA used as a preventative measure helps women having elective C-sections reduce blood loss safely.

Adverse Effects

TXA is often not associated with severe or incapacitating conditions. TXA is safe to use in a trauma environment because according to studies such as CRASH-2, it greatly lowers the risk of occurrence of myocardial infarction and has no impact on the rate of venous thromboembolism. While there is substantial proof that TXA lowers the need for blood transfusions during surgery, it is still unknown if it may also raise the risk of venous and arterial thromboembolism [48,49]. Furthermore, more investigation is needed to determine the significance of whatever anti-inflammatory properties TXA may have [50]. TXA usage is associated with seizures, it is recommended to use the lowest effective dose of TXA and to modify the dosage in response to clinical circumstances such as renal failure to reduce the risk of seizures [51].

Cautions

Although most of the TXA is excreted renally, effective research is lacking on the use of TXA in renally impaired individuals. However, it is recommended to adjust dosing and prudent administration in renal failure patients [51]. The liver-impaired patient does not need any modifications [26]. A study found that infants exposed to TXA through breast milk have low concentrations; hence, continuing the use of TXA in a nursing mother is recommended [52].

## Conclusions

This article discusses PPH, one of the prime causes of morbidity and mortality in pregnant females worldwide. It can occur after vaginal or C-section. PPH is dangerous for all women who carry a pregnancy over the 20-week mark and continues to be a prominent cause of maternal death in some parts of the world. Therefore, attending the final stage of labor is important for improving the prevalence of PPH.

TXA is a synthetic lysine derivative that inhibits fibrinolysis and stabilizes the preformed fibrin meshwork created by secondary hemostasis. TXA is FDA-approved for the treatment of menorrhagia and prophylaxis in hemophilia. However, off-label use has shown efficacy in various contexts, including total knee arthroplasty, spinal surgery, coronary artery surgery, gastrointestinal bleeding, epistaxis, traumatic brain injury, subarachnoid hemorrhage, micro-needling in melasma, dental extraction, painless lower urinary tract bleeding, complex kidney stones undergoing percutaneous nephrolithotomy, and scheduled liposculpture. Furthermore, TXA reduces blood loss and the requirement for blood transfusions in obstetric patients undergoing C-sections, as well as reduces mortality from PPH with good safety. However, the data on using TXA to prevent PPH is not as strong. TXA is a valuable option in managing obstetric bleeding, but its use should be tailored to individual patients and clinical circumstances. As with any medical particularization, it is critical to keep gathering and disseminating outcome data.
